# Proton minibeam radiotherapy: a review

**DOI:** 10.3389/fonc.2025.1580513

**Published:** 2025-07-21

**Authors:** Feng Yang, Junxiang Wu, Lucia Clara Orlandini, Heng Li, Xianliang Wang

**Affiliations:** ^1^ Department of Radiation Oncology, Sichuan Cancer Hospital & Institute, Radiation Oncology Key Laboratory of Sichuan Province, Affiliated Cancer Hospital of University of Electronic Science and Technology of China, Chengdu, China; ^2^ College of Nuclear Technology and Automation Engineering, Chengdu University of Technology, Chengdu, China; ^3^ Department of Radiation Oncology and Molecular Radiation Sciences, Johns Hopkins University, Baltimore, MA, United States

**Keywords:** proton minibeam radiotherapy, spatially fractionated radiotherapy, cancer treatment, implementation of pMBRT, radiobiological investigations

## Abstract

Radiotherapy plays a crucial role in cancer treatment. Spatially fractionated radiotherapy (SFRT) has garnered significant interest as a therapeutic strategy that delivers alternating regions of high and low radiation doses, thereby optimizing the therapeutic ratio by minimizing damage to adjacent normal tissues while achieving tumoricidal effects. Proton minibeam radiotherapy (pMBRT), a cutting-edge iteration within the SFRT paradigm, has attracted considerable attention owing to its purported benefits in dose distribution optimization, enhanced tumor control, and superior preservation of normal tissue. This manuscript presents an extensive evaluation of different applications of pMBRT, with a focus on the outcomes observed in preclinical research studies. Additionally, we explored the challenges faced in translating pMBRT from research to clinical practice, while also highlighting the significant potential this technique holds for the future of cancer treatment.

## 1 Introduction

**Figure 1:**
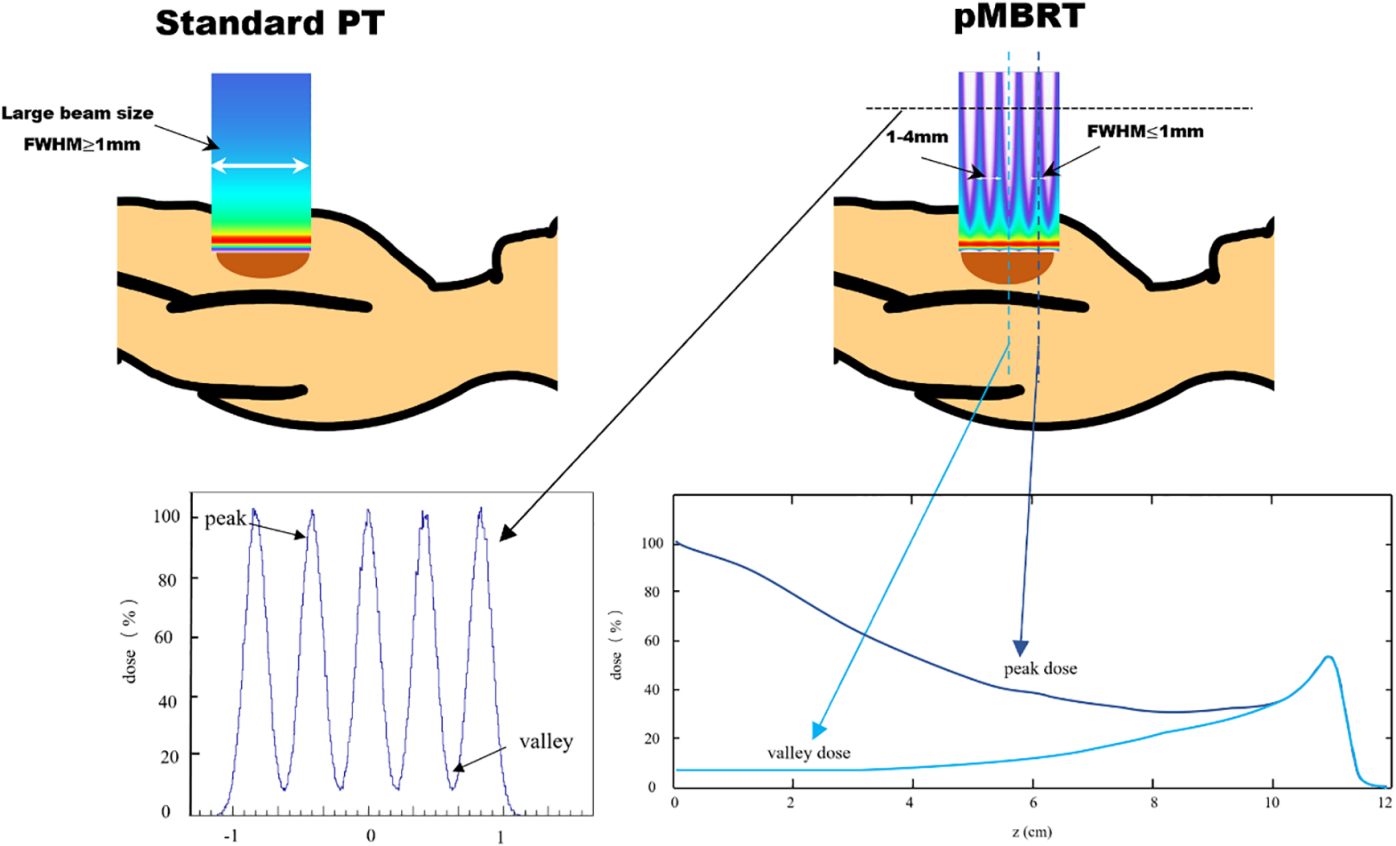
Comparison of standard proton therapy (PT) and proton minibeam radiotherapy (pMBRT).

Spatially fractionated radiotherapy (SFRT) has gained attention as a potential approach to improve the effectiveness of radiotherapy (1). Recent studies showed that SFRT could improve local control by leveraging spatially modulated dose patterns within the target volume, while reducing side effects through intentionally heterogeneous dose distributions in surrounding normal tissues (2–6). Minibeam radiotherapy (MBRT) is an innovative addition to the SFRT approach. Preclinical studies have demonstrated that MBRT significantly enhances the preservation of normal tissues, potentially enabling a safe escalation of the dose to the target while maintaining or improving tumor control (5, 7, 8). The MBRT field is composed of numerous sub-millimeter minibeams, with full width at half maximum (FWHM) dimensions generally ranging between 0.1 and 1 mm, and beam spacing typically between 1 and 4 mm. This configuration creates a pattern of alternating peak-dose and valley-dose regions (9). The comparison of standard proton therapy (left) and pencil beam microbeam radiation therapy (right) shows beam size differences, as illustrated in **Figure 1**. The peak-to-valley dose ratio (PVDR) varies depending on factors such as beam energy, minibeam size, center-to-center (CTC) distance, and depth, ranging from 1.2 to 13.3 in previous MBRT studies (10). However, PVDR can be well > 100 when using heavier ions (11), and when magnetic focusing is employed instead of collimators, magnetically focused minibeams exhibited a 20–60 times higher PVDR than mechanically collimated minibeams and yielded an increase in irradiation efficiency of up to two orders of magnitude (12, 13).

In particular, proton minibeam radiotherapy (pMBRT) (9) combines the dose deposition properties of protons with the protective potential of SFRT for normal tissue, offering additional advantages, thus spawning additional advantages (14). Firstly, the radiation dose beyond the Bragg peak is minimal, effectively protecting normal tissues located distal to the target. Given the high target dose intrinsic to MBRT, the potential of pMBRT to provide superior normal tissue sparing, as compared to X-ray-based MBRT, is particularly appealing. Secondly, due to proton Coulomb scattering within the tissue, the minibeams widen with depth such that irradiation configurations can be achieved that deliver a homogeneous dose to the tumor while preserving the peak and valley dose pattern in normal tissues proximal to the target volume. In spatially fractionated radiotherapy (SFRT), the design of multi-slit collimators significantly impacts dose distribution. For a 6.5 cm thick brass multi-slit collimator with five slits measuring 2 cm x 0.4 mm and a CTC distance of 4 mm, PVDR values range from 11.3 at the surface to 5 at 4 cm, until the spatial pattern fully dissipates at the Bragg peak (15).

According to the statistics of Particle Therapy Co-Operative Group (PTCOG), there are currently 137 proton centers in operation and 35 proton centers under construction worldwide, with more than 350, 336 patients treated with proton therapy by the end of 2023 (16). As an innovative technology, pMBRT has great potential to improve the radiotherapy outcome of large-volume tumors. Despite these advancements, it still faces considerable stumbling blocks in terms of technology implementation and clinical translation.

Given the rapid growth of proton therapy centers and increasing patient volumes, proton minibeam radiation therapy (pMBRT) holds significant promise for improving radiotherapy outcomes in large-volume tumors. This review comprehensively examines the implementation of pMBRT technology to date, summarizes key preclinical study results, and discusses its applications, challenges, and potential for clinical translation.

## 2 Implementation of pMBRT

A significant challenge in pMBRT is the generation of minibeams and its integration into clinical practice. pMBRT necessitates sub-millimeter beam sizes, whereas clinical proton therapy centers are typically configured to deliver either a uniform broad beam in passive scattering (17–19) or a narrow beam with FWHM dimensions in the range of several millimeters in pencil beam scanning (PBS) (20, 21). Therefore, there is a need to reduce the beam size further, which can be achieved by collimators or magnetic focusing (22).

### 2.1 Collimator

A minibeam collimator is a thick metal block with one or more small apertures that is placed at the end of a beamline. The apertures can take the shape of slits or holes (23–25). The characteristics of the apertures, including their dimensions, geometry, and positioning, play a critical role in shaping the resultant minibeam pattern. Extensive research has been dedicated to refining the collimator design for pMBRT, with successful applications observed in both passive scattering and PBS systems (5, 15, 26, 27).

To effectively reduce the valley dose and enhance the PVDR, the collimator must have substantial thickness, typically ranging from 7 to 10 cm, and should be positioned in close proximity to the target volume (28). The PVDR increases with decreasing collimator aperture size or increasing aperture spacing, but decreasing collimator aperture size or increasing aperture spacing leads to an inhomogeneous dose at the depth of the target volume. Therefore, a balance point has to be found to achieve the optimization goal. Lee et al. propose that for a 50 MeV proton beam, optimal parameters include a slit width of 0.3 mm and a 1 mm CTC spacing between adjacent slits (24). Guardiola et al. investigated various collimator materials to assess their differences. Their findings indicated that tungsten achieved the highest PVDR but also generated the most secondary neutrons. Brass, on the other hand, provided a balanced solution, offering a lower neutron yield with an acceptable PVDR. Additionally, brass was identified as a more cost-effective material due to its lower manufacturing and material expenses (28).

Minibeam collimators are generally custom-designed and remain static, featuring a fixed aperture arrangement that is optimized for particular applications. However, this rigidity limits their flexibility and reduces operational efficiency. To address this issue, Sotiropoulos et al. proposed a dynamic scanning collimator, featuring multiple adjustable brass blocks installed on a hexapod. This innovative design enables adjustments to both the position and orientation of the collimator, as well as the size of the apertures (29). Reaz et al. explored the feasibility of applying the moiré effect within a dual collimator system to create pMBRT dose profiles, offering a straightforward method to adjust the CTC spacing of the dose distribution. The angle between the two collimators significantly impacts the dose profile. CTC values ranging from 11.8 mm to 2.4 mm can be achieved by adjusting the dual collimator system angle from 10° to 50°. The dual multi-slit collimator system demonstrates significant versatility, being compatible with multiple beam types (such as X-rays and electrons) and adaptable to various SFRT techniques (2). Another practical challenge of collimators is the considerable impact of installation alignment uncertainties on dose distribution in pMBRT. Small deviations in manufacturing parameters such as tilt angle, slit width, spacing, and divergence angle, as well as variations in air gap, can significantly affect the dose distribution (30).

Collimators are a simple and straightforward option for generating proton minibeams. Nevertheless, employing these apertures significantly decreases the radiation beam fluence, leading to extended treatment times for patients. Furthermore, the interaction between proton beams and high atomic number materials generates secondary particles, including neutrons, which can also result in additional dose delivery to the patient.

### 2.2 Magnetic focusing technology

Quadrupole magnets are commonly used for beam focusing in clinical and experimental settings, and modern PBS beam lines often contain quadrupole magnets, which theoretically allow for the direct generation of minibeams by magnetic focusing. Proton minibeams generated by magnetic focusing have been presented at the SNAKE facility in Munich. This specialized facility achieves small beam sizes of about ∼ 10 μm (31). Since its maximum beam energy is 20 MeV, which is relatively low and has limited penetration capability, it can only be used for *in vitro* experiments and irradiation of skin models. Higher energies are still needed for the majority of clinical cases. The follow-up studies propose to upgrade the energy to 70 MeV (32). At present, the smallest beam sizes documented at clinical energy levels are approximately 4–5 mm FWHM. Therefore, a further reduction by an order of magnitude is required to achieve the beam size suitable for pMBRT.

**Figure 2:**
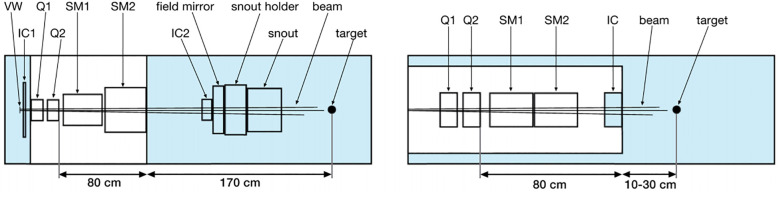
Schematics of the different nozzle geometries (35). Left: Model of the PBS nozzle at Orsay Proton Therapy Centre. Right: New, optimized nozzle design. VW, vacuum window; IC, ionization chamber; Q, quadrupole; SM, scanning magnet.

In fact, the currently available commercial PBS treatment heads are likely not able to produce sub-millimeter FWHM minibeams by magnetic focusing. This is because of two main factors: The first is scattering of beam particles (especially in long air gaps), which causes beam broadening (33). The second factor has to do with the fact that, in practice, beams have a non-zero emittance which prevents them from being perfectly focusable to a single point. In such conditions, the minimum focusing size scales approximately linearly with the focal length (34). In current commercial PBS systems, the focal length (typically > 2 m) is too large to allow sub-millimeter beam sizes to be achieved. Based on this, a new treatment nozzle design has been proposed, where the treatment nozzle consists of conventional beamline elements that are arranged more compactly compared with current PBS treatment heads, which can significantly reduce the air gap and focal length (35), show in **Figure 2**.

In summary, collimation and magnetic focusing are two distinct techniques used to control and direct beams. Collimation typically involves the use of physical apertures to shape and direct beams, which can result in some loss of beam intensity due to the blocking of certain parts of the beam. In contrast, magnetic focusing utilizes magnetic fields to converge and control the beam, which can be more efficient in terms of preserving beam intensity and allows for more precise control over the beam’s divergence and convergence. The key differences between these two methods are summarized in **Table 1**.

**Table 1:** Comparison of key differences between collimation and magnetic focusing.

Metric	Mechanical collimation	Magnetic focusing
Beam Spot Size	≥400 μm (limited by machining precision) (27)	Theoretical: 1–2 μm; Clinical measurement: 5–10 μm
Beam Utilization Rate	≤10% (majority of protons blocked) (36)	~100% (no material obstruction) (33)
Secondary Neutron Yield	less than 1% to the patient dose (28)	Negligible
Efficiency (33)	Hardware replacement required (time-consuming, inflexible)	More effective than collimator
Depth Adaptability	Compatible with 100–230 MeV energy range (27)	Requires special design for high-energy protons (>150 MeV) (15, 35)
Implementation at existing facility	Yes	No

## 3 Radiobiological investigations with preclinical studies

The ability of pMBRT to protect normal tissue while enhancing therapeutic results has been shown in various cellular and animal studies (37–39). Preclinical results indicate that pMBRT effectively preserves cerebral functions and reduces neuroinflammation and toxicity compared to conventional proton therapy, demonstrating its potential for improved therapeutic outcomes (37, 38). Studies on human skin and mouse ear models also found reduced skin toxicity, and pMBRT has the potential to significantly enhance normal tissue tolerance to peak doses in the range of 100–150 Gy (31, 40–43). In addition, experiments on rats carrying gliomas showed that pMBRT resulted in good tumor control rates (44). When minibeam was used in place of standard wide-beam proton therapy irradiation, the long-term survival rate increased by a factor of 3 (5, 26). Bertho et al. examined the tissue-sparing benefits of pMBRT on mice using a clinical machine and standard dose rates. Compared to conventional proton therapy, pMBRT-induced lung changes were more localized and less severe (7).

Understanding the mechanisms behind the tissue-preserving effects of spatial segmentation is crucial for identifying optimal irradiation parameters for clinical application. Through high-resolution spatiotemporal analysis, damage heterogeneity and its dynamic changes at the single-cell level were revealed for the first time in realistic 3D tissue models (41). PVDR is considered a biologically significant parameter, with optimal normal tissue preservation occurring when the valley dose is minimized and the PVDR is high (28, 45). This phenomenon could be explained by the migration of healthy cells from non-irradiated neighboring tissue into the irradiated and damaged areas, which helps repair the affected region and enhances tolerance (46). In addition, there are indications that there may be a link to off-target effects as well as additional cell signaling interactions (47, 48). Dose volume effects may also play a role, as smaller irradiated tissue volumes are associated with higher maximum tolerated doses. This repair process may be supported by the micro-rapid tissue repair effect, which refers to the rapid regeneration of capillaries promoted by intact antigen cells within the valley area and highlighting how localized tissue dynamics contribute to overall tolerance and recovery following irradiation (49, 50).

Radiotherapy’s impact on the immune system is dose-dependent, with low doses enhancing tumor vascular normalization and T cell infiltration (51), while high doses eliminate immunosuppressive cells, releasing damage-associated molecular patterns to activate anti-tumor immunity (52). SFRT can overcome the radioresistance of hypoxic regions within tumors by delivering high peak doses, enhancing the immune response and modulating the tumor microenvironment (53). Preclinical evidence supports SFRT’s potential for tumor regression and systemic immune activation (54). Clinical studies have shown that combining SFRT with immune checkpoint blockade improves patient outcomes, highlighting a synergistic approach to cancer therapy (55). In mouse models, targeted irradiation of tumor volumes stimulates a potent immune response, particularly enhancing CD8^+^ T cell activity (56). For patients with recurrent, unresectable tumors, novel radiation modalities like carbon ion and proton therapy have demonstrated efficacy in inducing abscopal effects, showcasing promise in safety and tolerability (57). The immune system may be central to the therapeutic effects of pMBRT (58). In comparison to glioma-bearing rats, it was found that only animals with strong immune ability respond to MBRT treatment, while nude rats with immune deficiency do not respond (59). Recent studies suggest that the combination of ultra-high dose rates (FLASH effect) with spatial fractionation could further enhance normal tissue sparing while maintaining tumor control, offering a promising avenue for future clinical applications (60, 61).

Recent preclinical studies on proton minibeam radiation therapy (pMBRT) have explored various animal models and tumor types, demonstrating significant reductions in local tissue toxicity while maintaining or even enhancing tumor control efficacy compared to conventional radiotherapy. A summary of the latest animal models, tumor types, and efficacy data in preclinical pMBRT studies is provided in **Table 2**.

**Table 2:** Summary of animal models, tumor types, and efficacy data in preclinical pMBRT studies.

Model	Model type	Study subject	Beam energy (MeV)	Beam size(mm)	PVDR value(maximum)	Stage	Reference
Phantom	Simulation	–	100	0.4	22.3 ± 0.2	Monte Carlo simulation	Cavallone et al., 2022 (62)
			100	–	1000		Schneider et al., 2020 (35)
			105	–	60 ± 3		Prezado & Fois, 2013 (14)
			100	–	772 ± 2		Schneider, 2020 (25)
			150	0.4	6.87 ± 0.05		Lin et al., 2025 (63)
			105 1GeV	0.7	15.6 ± 0.8 23.2 ± 1.1		Prezado & Fois, 2013 (14)
			100	0.4	10 18		Guardiola et al., 2017 (28)
			–	–	30.96		Kim et al., 2022^16]^
	Mearsure	–	123 150	0.4	16.0 ± 2.8 11.3 ± 2.5	Monte Carlo Simulation + Measure	De Marzi et al., 2018 (15)
			100	0.4	8.42	Measure	Lin et al., 2024 (64)
Animal Models	Fischer 344 rat	High-grade glioma (RG2)	100	0.4	6.5	Preclinical	Prezado et al., 2018 (44)
		Normal brain tissue	100	0.4	–	Preclinical	Prezado et al., 2017 (39)
		Glioma (F98)	100	0.4	–	Preclinical	Lamirault et al., 2020 (65)
	Mouse	Skin toxicity	20	0.018~0.52	–	Preclinical	Girst et al., 2015 (40)
				0.01 + 0.05	–	Preclinical	Zlobinskaya et al., 2013 (31)
				0.18	–	Preclinical	Girst et al., 2016 (42)
				0.095-0.83	>540	Preclinical	Sammer et al., 2019 (43)
Human Models	3D human skin model	Normal skin	20	0.066 0.408 0.92	∞ 45 1.35	*In vitro*	Scherthan et al., 2022 (41)
	In silico simulation	Brain metastases、 Liver metastases、 Lung metastases、 Glioma/Meningioma patients	235	0.4	24.3 ± 0.5	Computational	Ortiz et al., 2023 (66)

## 4 Treatment planning studies

Promising results reported in preclinical studies have encouraged efforts for the clinical stage. Several theoretical investigations have been conducted focusing on treatment planning for pMBRT. These studies have explored various beam models, each representing distinct clinical and experimental beamline configuration and ideal theoretical sources were simulated using the Monte Carlo simulation (33). A specialized Monte Carlo dose calculation method was developed to analyze treatment planning in high-grade glioma and meningioma cases (67). To further amplify the therapeutic advantages of proton minibeams, Ortiz et al. investigated the potential of integrating pMBRT with arc therapy (68). A study evaluated the feasibility of using pMBRT to treat four clinical cases that were previously treated with stereotactic radiotherapy (SRT). The findings demonstrated that pMBRT offered comparable or improved target coverage relative to SRT, despite employing fewer fields.

**Figure 3:**
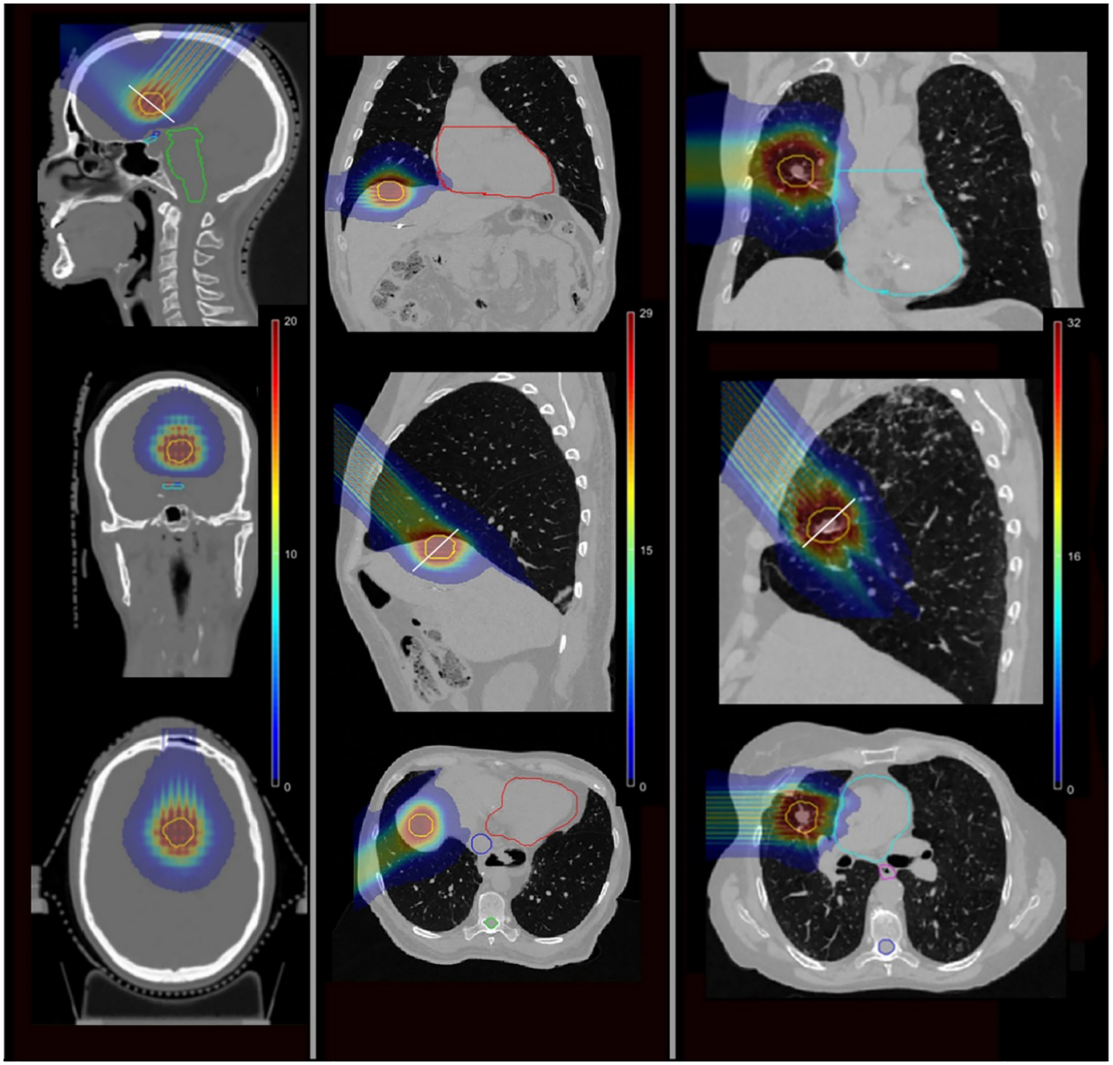
The isodose colorwash of pMBRT plans for different cases, the target is displayed in yellow (68). Left: a brain case. Middle: a liver case. Right: a lung case.

The spatial variability of relative biological effectiveness (RBE) plays a crucial role in optimizing therapeutic protocols (69). The RBE value ranged from 1.05 to 1.4 in normal tissues, while in the target, it varied between 1.07 and 1.1, which may confer a therapeutic advantage by potentially enhancing the cytotoxicity of the treatment (67). Comparative dosimetry studies found that compared with traditional SRT, pMBRT was related to the decrease of the average normalized tissue dose at 2 Gy-fractions. In the lung, the corresponding value of pMBRT was 1.7 Gy [RBE], and that of the traditional SRT was 2.6 Gy [RBE]. In the liver, the corresponding value of pMBRT was 1.0 Gy [RBE], and that of the traditional SRT was 3.8 Gy [RBE] (66). **Figure 3** illustrates the dose distribution of pMBRT in different tumor sites. Loap et al. conducted a simulation of single-fraction ventricular tachycardia ablation, showing that pMBRT could achieve uniform coverage of an arrhythmogenic cardiac region. pMBRT could theoretically lower the risk of late-onset pulmonary and breast fibrosis, as well as cardiac toxicity (70).

In pMBRT dosimetry, two inherent challenges significantly predispose the process to errors: the extremely small beam sizes used and the necessity to accurately resolve peaks and valleys across a large dynamic range. These challenges necessitate precise measurement and modeling techniques to ensure accurate dose distribution. Accurate dosimetry and quality assurance programs are crucial for the safe and effective delivery of pMBRT. In pMBRT dosimetry, traditional methods often fail to provide accurate results due to the extremely small beam sizes used. One study addressed this limitation by developing a novel equation for calculating the radiation quality correction factor in pMBRT. This equation was specifically designed to account for the unique challenges posed by minibeams geometries and dose distribution patterns. When applied, the correction factor revealed that the dose was significantly overestimated by approximately 10% in both open field and minibeam field cases for X-rays and proton beams (71). Lin et al. used a high-resolution dosimetry instrument to verify pMBRT. When properly calibrated and corrected, highly consistent dose distributions can be obtained under various conditions (64).

Before pMBRT can be considered for routine clinical application, it is essential to establish a set of reference parameters that accurately characterize and quantify its effectiveness. pMBRT is usually quantified in terms of PVDR and FWHM. One study improved an existing parameter of pMBRT, CTC distance, and defined the amount of transmission for collimated pMBRT irradiation. The CTC distance directly affects the transmission efficiency and overall dosimetric accuracy (28). For a given beam FWHM, increasing the beam CTC spacing can result in a lower valley dose. Additionally, a larger CTC allows for achieving a specified field size with fewer beams. Currently, PVDR is optimized by varying the machine configuration (e.g., collimator, spot size, thickness, and CTC). A new approach to pMBRT treatment planning has been developed to co-optimize the plan by maximizing the PVDR while ensuring a reasonable 3D dose distribution, which balances high PVDR values for effective target dosing with constraints on healthy tissue exposure (72).

## 5 Discussion and conclusion

pMBRT is a novel approach within the realm of SFRT, offering potential advantages over conventional proton therapy for treating large tumors. Preclinical trials have shown promising results, with significant normal tissue sparing and effective tumor control. These studies represent a significant advancement toward the clinical application of pMBRT. However, its clinical benefits have yet to be conclusively demonstrated (67, 73).

The development of appropriate equipment for the clinical implementation of pMBRT remains an ongoing challenge. For the optimal delivery of pMBRT, minibeams generated through magnetic focusing scans would be ideal, despite the proven effectiveness of mechanical collimators in experiments with clinically relevant beam energies. Generating the beam in this way will allow for maximum irradiation flexibility and reduced secondary particle contamination. Additionally, the magnetically focused scanning minibeam allows for improved beam utilization and high dose rate irradiation, as it converts the entire initial beam into minibeams without blocking part of it, unlike mechanical collimators, which thereby increases the attainable dose rates and delivery efficiency. The benefits of high-dose-rate irradiation are at least threefold. Firstly, it improves treatment efficiency. Secondly, it prevents the blurring of peak and valley doses due to organ motion (74). The third is the combination of FLASH technology (an emerging technique delivering ultra-high dose rate radiation (≥40 Gy/s) within milliseconds) to increase patient benefit further (60).

Current treatment planning systems do not yet support sub-millimeter proton beam dose calculations. Monte Carlo simulation serves as an effective tool for designing and optimizing the generation of minibeams under clinical conditions. To accurately simulate collimated minibeams, a detailed simulation of the collimator is essential. However, these simulations can be time-consuming and potentially slowing down the overall computation of the treatment plan (36). In contrast, the simulation of a magnetically focused minibeam is much faster since only the beam-air interaction has to be taken into account at the upper end of the target area. In this case, magnetic focusing is the preferred method for 3D optimization of the dose for pMBRT.

Given the distinct dosimetric characteristics of pMBRT, the irradiation setup has a significant impact on dose accuracy, due to its extremely small beam sizes and complex dose distribution patterns. Research has shown that even a minor misalignment between the beamline and the collimator inlet can significantly alter the lateral dose distribution, resulting in a decrease in PVDR and an increase in FWHM by up to 50% (30). The dose distribution can be impacted by unexpected setup variations and uncertainties in collimator manufacturing. These factors may skew the correlation between dosimetric measurements and biological endpoints (70). For plans with multiple fields, alignment constraints can be further alleviated using an orthogonal approach, which is more tolerant of the accurate placement of overlapping peak and valley regions in different fields. Additionally, the requirements for shot field-to-shot field positioning can be relaxed due to enhanced alignment technologies, permitting greater flexibility while maintaining treatment accuracy (26).

The predictive modeling of RBE in pMBRT requires complicated computing techniques such as Monte Carlo simulations. These methods account for the complex interaction between proton beams and biological tissue. Simulations may help estimate RBE, though RBE remains an inherently empirical quantity derived from experimental measurements for specific radiobiological endpoints (24, 75). The validation of pMBRT predictive models is achieved through the application of preclinical animal models, with a comprehensive evaluation of the ensuing effects on both normal and tumor tissues (39, 44). This evaluation not only confirmed the accuracy of the RBE predictions but also clarified the therapeutic potential of pMBRT in clinical oncology. The findings from these studies are valuable for promoting our understanding of the complex interactions between proton beams and various tissue types, thus supporting the biological basis of clinical treatment plans.

The first MBRT treatments conducted to date have exclusively utilized X-rays. Kundapur et al. were the first to apply X-ray MBRT in treating brain tumors in dogs (76). Subsequent comparative studies between MBRT and standard radiation treatment revealed potential advantages of MBRT in brain tumor management, particularly in terms of tumor control and preservation of normal tissue structures (77). Grams et al. reported the first clinical implementation of MBRT, utilizing orthovoltage apparatus and custom-designed tungsten alloy collimators to generate peak-dose and valley-dose regions within the tumor. They employed 3D-printed collimator fixators to mitigate the potential impact of patient movement on dose delivery, successfully administering MBRT to two patients (78). Compared to X-ray-based MBRT, pMBRT displays unique radiobiological properties (31, 39, 42, 79), with the potential to achieve uniform dose distributions at depth while preserving modulation capabilities at the beam’s entrance (9). While pMBRT has not yet been clinically implemented, efforts towards its clinical translation have been extensively discussed in preceding sections.

In conclusion, pMBRT is an innovative technology whose unique dose distribution characteristics and preclinical outcomes provide the rationale for further research and clinical trials. Preclinical studies have demonstrated its efficacy in tumor control and normal tissue preservation across various tumor models, including brain and skin cancers. However, the clinical translation of pMBRT is currently hindered by technical challenges, such as the development of specialized collimators and treatment planning systems, as well as the need for further biological validation to establish its safety and efficacy. Future research should focus on optimizing these technical aspects, elucidating the radiobiological mechanisms underlying pMBRT, and conducting clinical trials to evaluate its therapeutic potential in cancer treatment.
